# State Medical Service

**Published:** 1918-11-30

**Authors:** Harold Burrows

**Affiliations:** Moorend Park, Charlton Kings, Cheltenham.


### THE EDITOR'S LETTER-BOX.

[Correspondence on all subjects is invited, but we cannot in any way be responsible for the opinions expressed by our correspondents, who must give their name and address as a guarantee of good faith, but not necessarily for publication. Correspondents are reminded that brevity of style and conciseness of statement greatly facilitate early insertion.]

### State Medical Service.

*To the Editor of* THE HOSPITAL.

SIR,—I have read with interest Col. Maurice's articles on "A State Medical Service." Thoughtful people will agree that this involves perhaps the most urgent domestic problem of to-day. Personally, I am one of those who believe that, if the war has taught us a true appreciation of the economic value of an efficient Medical Service, and if the politicians will play their part fairly in the public interest, the entire cost of the war both in lives and in money, gigantic as this cost has been, will be redeemed within a few years.

Unfortunately, there seems to be a profound distrust among many medical men as to the outcome of any political measures that may be taken to remedy the existing slipshod and chaotic supply of preventive and therapeutic medicine and surgery to the public. Such distrust is not entirely unwarranted by past experience. It is feared that any State Medical Service likely to emanate from Parliament will be a cheap and nasty one, and will furthermore be too much under lay control. Certainly it is essential, if the best brains of the country are to be attracted and efficiency is to be maintained, that a State Medical Service must offer good remuneration to those who are worthy of it, encouragement and advancement for those who devote themselves to other branches than administration, and control of the *personnel* and establishment by those who are in a position to perform proper judgments. No layman can estimate correctly the capacity of a technical expert. Therefore in a State Medical Service all advancement must be controlled from within the profession. If the high offices are to be held as political prizes, a State Medical Service will be handicapped by disrepute from the time of its birth.—Yours, etc., HAROLD BURROWS.

Moorend Park, Charlton Kings, Cheltenham.
November 24, 1918.

